# Trajectory analysis quantifies transcriptional plasticity during macrophage polarization

**DOI:** 10.1038/s41598-020-68766-w

**Published:** 2020-07-23

**Authors:** Serena X. Liu, Heather H. Gustafson, Dana L. Jackson, Suzie H. Pun, Cole Trapnell

**Affiliations:** 1 Department of Genome Sciences, University of Washington, Seattle, WA 98195 USA; 2 Department of Bioengineering, University of Washington, Seattle, WA 98195 USA; 3 Present Address: Ben Towne Center for Childhood Cancer Research, Seattle Children’s Research Institute, Seattle, WA 98101 USA

**Keywords:** Cell biology, Molecular biology, Transcriptomics

## Abstract

In recent years, macrophages have been shown to be tremendously plastic in both in vitro and in vivo settings; however, it remains unclear whether macrophages retain any persistent memory of past polarization states which may then impact their future repolarization to new states. Here, we perform deep transcriptomic profiling at high temporal resolution as macrophages are polarized with cytokines that drive them into “M1” and “M2” molecular states. We find through trajectory analysis of their global transcriptomic profiles that macrophages which are first polarized to M1 or M2 and then subsequently repolarized demonstrate little to no memory of their polarization history. We observe complete repolarization both from M1 to M2 and vice versa, and we find that macrophage transcriptional phenotypes are defined by the current cell microenvironment, rather than an amalgamation of past and present states.

## Introduction

Cellular plasticity broadly refers to cells’ ability to assume different phenotypic identities. While cellular plasticity is perhaps best known as a canonical feature of embryonic differentiation in early development, it is also crucial for enabling differentiated cells to respond dynamically to changing microenvironments, as in the case of immune cells redirecting their function in response to different extracellular signals^1^.

Macrophages perform a wide variety of crucial, and sometimes contradictory, functions. While “pro-inflammatory” activities like fighting off infections and “anti-inflammatory” activities like wound-healing traditionally have been attributed to M1 and M2 macrophage subsets, studies of macrophages in both in vitro and in vivo contexts suggest that macrophages are phenotypically plastic and may shift states in response to environmental changes. Introducing a combination of CpG oligodeoxynucleotides (TLR9 agonist) and anti-interleukin-10 receptor antibody (IL-10 signaling antagonist) to tumor-associated macrophages in vivo triggered a phenotypic switch from M2-like to M1-like^2^. Tissue-resident macrophages also display a similar plasticity: peritoneal macrophages that were transferred to the lung adopted a lung-specific phenotype, down-regulating peritoneal macrophage-specific genes and up-regulating lung macrophage-specific genes^3^. In addition, treating macrophages with different cytokines sequentially in vitro triggered corresponding changes in the expression of canonical murine macrophage markers like iNOS and arginase, as well as changes in the panel of cytokines secreted by the stimulated macrophages^4^.

Macrophages that have been polarized in vitro, reprogrammed in situ, or engineered with genome editing tools are a promising avenue for cell-based therapeutics^5^. Macrophage dysfunction has been implicated in a wide array of diseases, including asthma^6–8^, obesity^9–12^, cancer^13–17^, and atherosclerosis^14,18,19^. In many of these cases, macrophages are thought to play a key role in disease pathogenesis and are considered a promising therapeutic target. Phenotypic shifts from M2-like to M1-like, or conversely from M1-like to M2-like, have been linked to disease outcome in cancer and obesity^2,20–25^. Moreover, efforts to treat disease by reprogramming macrophages or by transplanting engineered macrophages have shown some success in cancer^2,20–23^ and hereditary pulmonary alveolar proteinosis (hPAP)^5^. Thus, developing a better understanding of the molecular mechanisms controlling phenotypic shifts between M1-like and M2-like states could give insight into disease etiology and could lead to the discovery of novel therapeutic targets.

While macrophage plasticity is now well-established, it remains unclear the extent to which macrophages retain any memory of past phenotypic states and whether a cell’s history might impact its future response to environmental polarization cues. For instance, a dynamical systems view of cellular differentiation posits that cells undergoing molecular state transitions are “attracted” to specific positions along the continuum of molecular phenotypes they might adopt, and that these “attractors” correspond to classically defined cell types^26,27^. It is conceivable that polarization drives macrophages to such attractor states, resulting in stable and self-sustaining activation. Alternatively, stimulation to M1- or M2-like states may be transient and entirely dependent on the continuous presence of chemical cues in the microenvironment. Furthermore, it is also unclear whether the mechanism of macrophage plasticity operates on the level of individual cells (each cell undergoing a transition from old to new phenotype) or on the level of cell populations (subpopulations displaying the old phenotype being replaced by subpopulations displaying the new phenotype). In vitro models of macrophage polarization offer a controlled setting in which to begin investigating the molecular drivers of cellular phenotypic plasticity. Since we culture macrophages under conditions with little to no cell growth, observed changes in cell phenotype can be attributed to plasticity at the level of individual cells.

Here, we investigate the extent to which macrophages’ previous polarization affects their response to subsequent repolarization. To this end, we first polarized murine bone marrow-derived macrophages to M1 and M2 states in vitro, then observed polarized macrophage responses to either the removal of extrinsic cytokine stimulation altogether or repolarization to the opposite polarization state via a switch in polarizing cytokines. Both the initial polarization trajectory and the subsequent repolarization were characterized using bulk time-course transcriptomic sequencing. We find that in vitro mouse macrophages adopt phenotypes directed primarily by their current microenvironment, irrespective of previous polarization state.

## Results

### Cytokine stimulation drives synchronous and homogenous macrophage polarization in vitro

**Figure 1:**
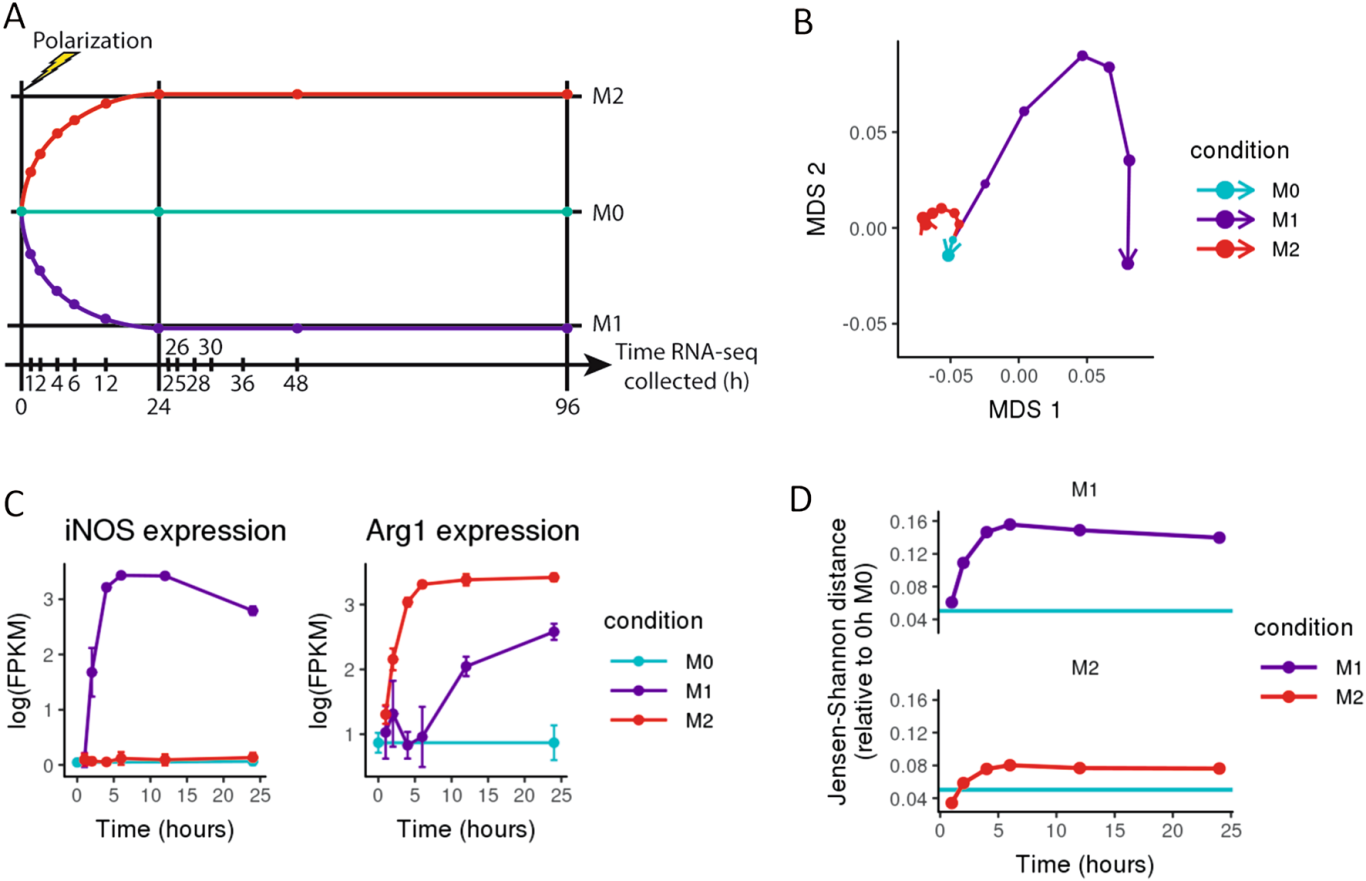
In vitro polarized macrophages display distinct M1 and M2 phenotypes. (**A**) Schematic of experimental design, where each point represents a collected sample, and each colored trajectory represents a different treatment condition. Murine bone marrow-derived macrophages were treated with cytokines to induce polarization (IFN-γ + LPS for M1, IL-4 for M2); M0 was maintained in base media as an unpolarized control (n = 3). (**B**) Multidimensional scaling (MDS) plot of RNA-seq expression (averaged across three replicates for each sample); points representing each sample are connected in order of collection, with point size representing the time spent (in hours) in culture. Macrophages treated with IFN-γ + LPS (M1) and IL-4 (M2) follow distinct polarization trajectories. (**C**) Gene expression in log(FPKM) of *iNOS* (a marker gene for M1 polarization) and *Arg1* (a marker gene for M2 polarization) over time. (**D**) Jensen–Shannon distance (JSD) between the RNA expression profiles of M1- and M2-polarized macrophages and the 0 h M0 control. As a baseline for expected global expression differences between samples, the teal line shows the average JSD between all later M0 controls (collected at 24 h, 48 h, and 96 h) and the original 0 h M0 control sample.

In order to measure the full transcriptomic profiles of in vitro polarized macrophages, we treated murine bone marrow-derived macrophages (BMDMs) with either interferon-γ (IFN-γ) and lipopolysaccharide (LPS) to induce an M1 polarization state or interleukin-4 (IL-4) to induce an M2 polarization state. Macrophages were harvested at 1, 2, 4, 6, 12, and 24 h after the addition of cytokines and profiled using bulk RNA-seq (Fig. 1A). We visualized a global transcriptional “trajectory” of these timepoints using multidimensional scaling (MDS), which showed that macrophages embark on distinct programs of gene regulation upon polarization to M1 or M2 (Fig. 1B). To identify differentially expressed genes (DEGs), we compared the 0 h M0 timepoint to each M1 and M2 timepoint using Cuffdiff. As measured by the number of differentially expressed genes, at 24 h, M1 cytokines elicited a stronger transcriptional response (5,387 genes) than M2 cytokines (2,811 genes) (Fig. S1). These included previously defined M1 (*iNOS*) and M2 (*Arg1*) gene markers (Fig. 1C & S2), confirming that our in vitro polarized macrophages developed distinct and expected M1 and M2 phenotypes.

To globally quantify the transcriptomic differences between M1- and M2-polarized macrophages and unpolarized M0 macrophages, we computed the Jensen–Shannon distance (JSD), a measure commonly used to assess transcriptomic similarity^28^, between each polarized sample and the 0 h M0 sample. We then compared these distances to the JSD between the later M0 controls (24 h M0, 48 h M0, and 96 h M0) and 0 h M0 (Fig. 1D), which establishes the expected variation between unpolarized, cultured macrophages over the time scale of the experiment. Both M1 and M2 macrophages trend towards equilibrium JSD values which are higher than the JSD between different M0 timepoints, reflecting stable polarized phenotypes that are distinct from the unpolarized control. That M1 macrophages are more distant from M0 than M2 cells are by this measure is consistent with the greater number of DEGs between M1 and M0 than between M2 and M0.

In principle, cytokines could elicit heterogeneous or asynchronous responses in individual macrophages, so we analyzed a subset of these timepoints with single-cell transcriptome sequencing. M1- and M2-polarized macrophages formed coherent clusters that were clearly separated from each other and unpolarized cells, with no intermixing of cells from different time points, suggesting that this in vitro system drives macrophage polarization in a largely synchronous and homogeneous manner (Fig. S3A). The in vitro cultured macrophages also exhibited very low expression of proliferation markers, particularly in the M1 condition, suggesting a low level of cell growth (Fig. S3B,C).

### Polarized macrophages return to baseline upon removal of extrinsic cytokines

**Figure 2:**
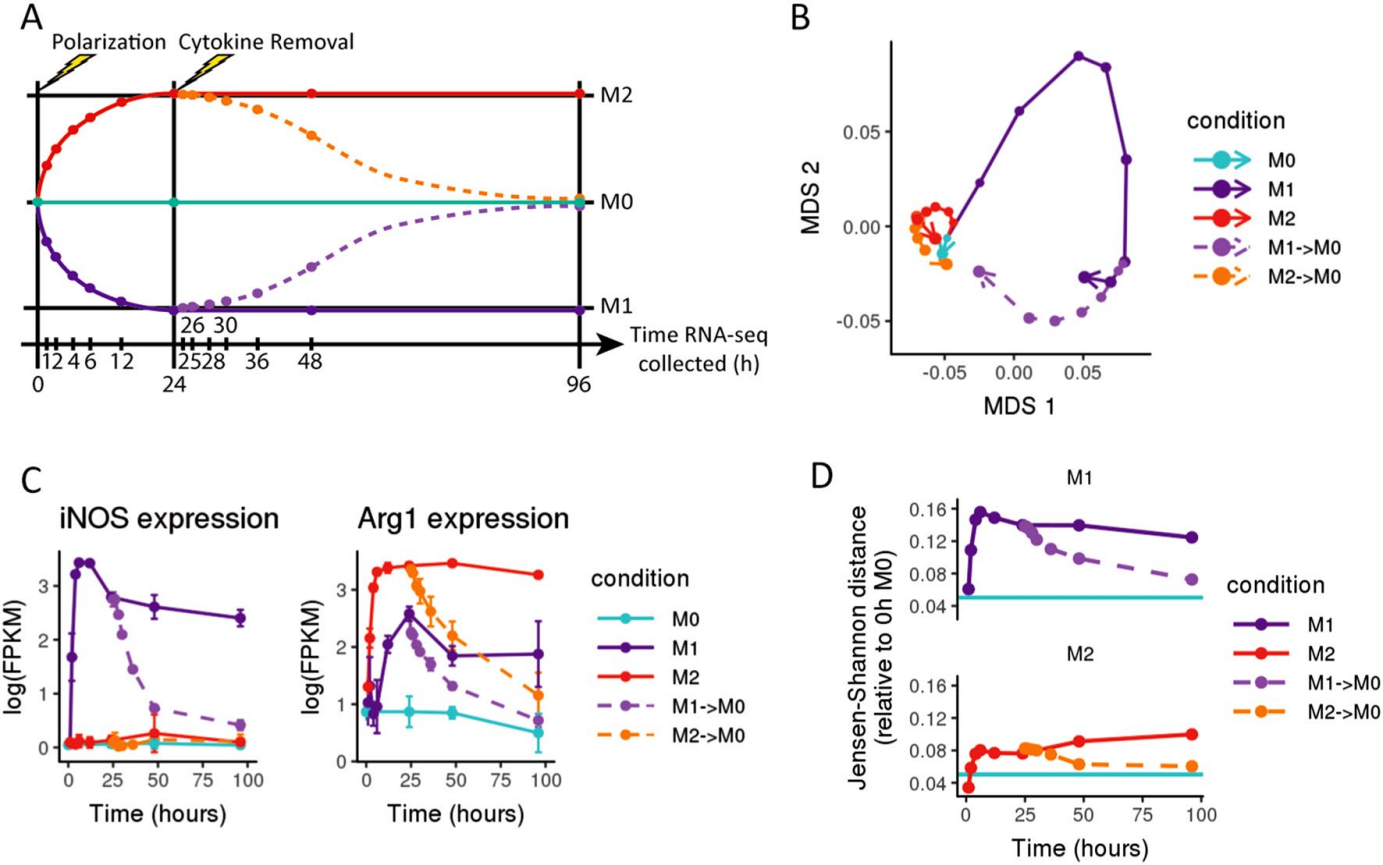
Polarized macrophage phenotypes are transient. (**A**) Schematic of experimental design, where each point represents a collected sample, and each colored trajectory represents a different treatment condition. Murine bone marrow-derived macrophages were treated with cytokines to induce polarization (IFN-γ + LPS for M1, IL-4 for M2); M0 was maintained in base media as an unpolarized control. After 24 h, cells were washed to remove cytokines and then cultured in base media with no polarizing cytokines (n = 3). (**B**) Multidimensional scaling (MDS) plot of RNA-seq expression (averaged across three replicates for each sample); points representing each sample are connected in order of collection, with point size representing the time spent (in hours) in culture. After the removal of polarizing cytokines, the expression profiles of previously polarized macrophages follow trajectories that trend towards the M0 baseline. (**C**) Gene expression in log(FPKM) of *iNOS* (a M1 marker gene) and *Arg1* (a M2 marker gene) over time. While marker gene expression increases with initial polarization, it drops sharply upon the removal of polarizing cytokines and falls to near baseline levels after 72 h in base media. (**D**) Jensen–Shannon distance (JSD) between the RNA expression profiles of polarized and depolarized macrophages compared to the 0 h M0 control. As a baseline for expected global expression differences between samples, the teal line shows the average JSD between all later M0 controls (collected at 24 h, 48 h, and 96 h) and the original 0 h M0 control sample. While JSD initially increases as polarizing macrophages develop distinct mRNA expression profiles and diverge from the unpolarized baseline, removal of polarizing cytokines triggers a reversion towards the baseline.

We then wished to explore whether M1- and M2-polarized macrophages could maintain their polarized phenotypes in the absence of extrinsic cytokines in the media, or whether M1 and M2 macrophages would revert back to an unpolarized state without continuous external cytokine stimulation. To investigate this question, M1- and M2-polarized macrophages were washed and then returned to a basic media without any polarizing cytokines (Fig. 2A). Separately, we maintained M1 and M2 macrophages in culture with the original polarizing cytokines as a control. In the absence of continued cytokine exposure, we found that previously polarized macrophages quickly revert to a state that resembles the baseline unpolarized phenotype, in terms of both the expression of individual M1/M2 polarization marker genes and the cells’ overall transcriptomes (Fig. 2B,C). After 72 h in cytokine-free media, we detected 2,307 differentially expressed genes between M1 → M0 macrophages and 0 h M0 macrophages (as compared to 4,732 DEGs for the 72 h M1 control) and 2,038 differentially expressed genes between M2 → M0 macrophages and 0 h M0 macrophages (as compared to 3,824 DEGS for the 72 h M2 control) (Fig. S4). JSD between depolarizing M1 and M2 macrophages and 0 h M0 also trends down towards the baseline JSD value between different M0 timepoints (Fig. 2D). Most M1 and M2 marker genes returned to levels very similar to the M0 cells, although there were some exceptions. For example, following removal of M1 cytokines, while *Tnf* was reduced to 36.7% of its peak expression levels and 62.7% of equilibrium 96 h M1 levels, *Tnf* expression remained slightly elevated relative to M0 cells at 96 h (Fig. S5A). Similarly, while *Retnla* expression levels drop to 55% of their 24 h M2 levels in 96 h M2 → M0 cells, the gene is still expressed at higher levels than in M0 cells (Fig. S5B). These results suggest that most genes upregulated as part of M1 or M2 polarization do not remain expressed at maximal levels in the absence of extrinsic cytokine stimulation, often returning to their pre-polarization levels.

### Polarized macrophages exposed to new cytokines undergo repolarization to a new phenotype

**Figure 3:**
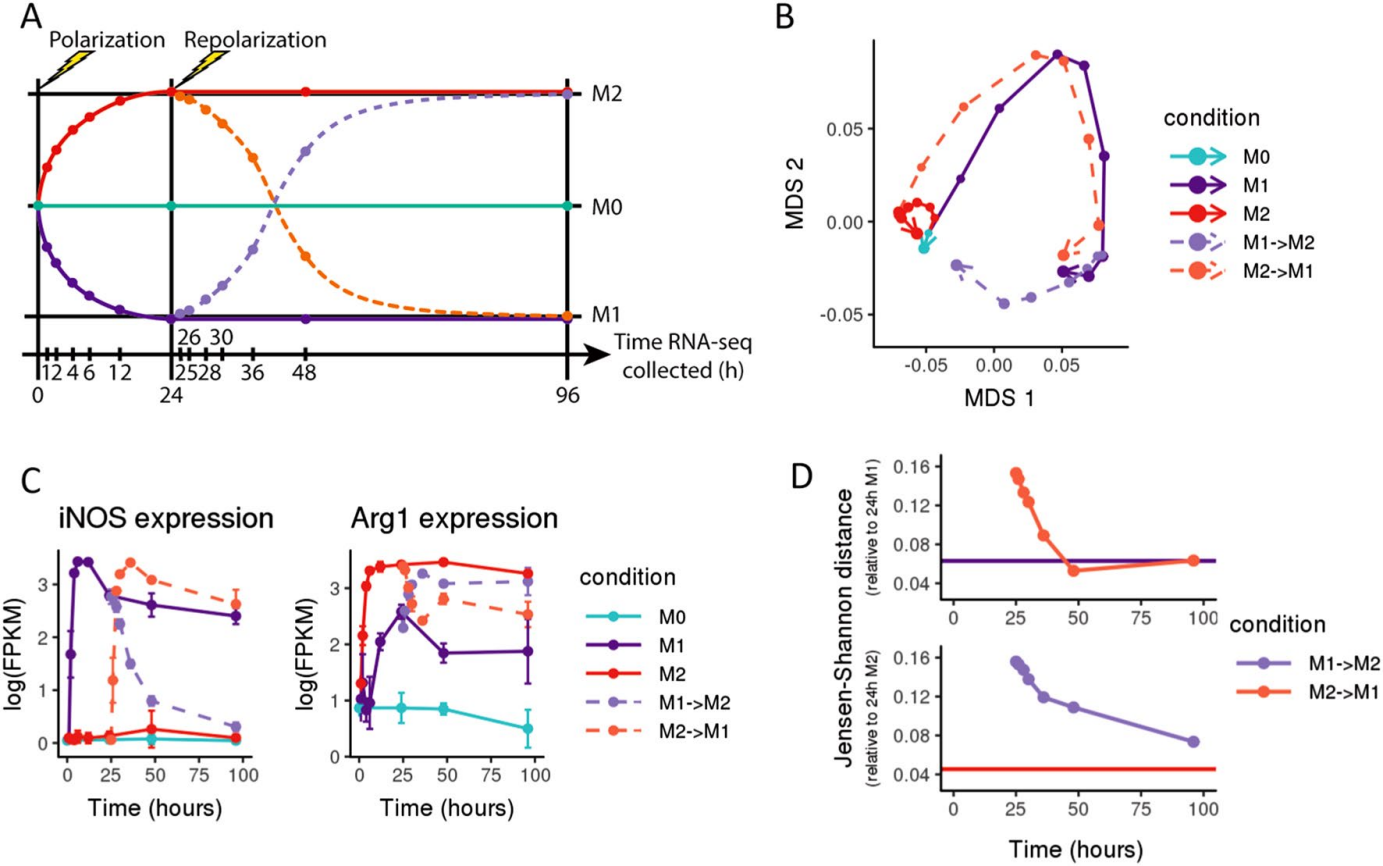
Macrophages convert between polarized states in response to cytokine treatment. (**A**) Schematic of experimental design, where each point represents a collected sample, and each colored trajectory represents a different treatment condition. Murine bone marrow-derived macrophages were treated with cytokines to induce polarization (IFN-γ + LPS for M1, IL-4 for M2); M0 was maintained in base media as an unpolarized control. After 24 h, cells were washed to remove cytokines and then cultured with the alternate set of polarizing cytokines (M1 switched to M2, and vice versa) (n = 3). (**B**) Multidimensional scaling (MDS) plot of RNA-seq expression (averaged across three replicates for each sample); points representing each sample are connected in order of collection, with point size representing the time spent (in hours) in culture. After switching polarizing cytokines, the expression profiles of previously polarized macrophages follow trajectories that trend towards the new polarized state. (**C**) Gene expression in log(FPKM) of *iNOS* (a M1 marker gene) and *Arg1* (a M2 marker gene) over time. Switching the polarizing cytokines induces reduced expression of the previous marker gene and increased expression of the marker associated with the new polarization state. (**D**) Jensen–Shannon distance (JSD) between the RNA expression profiles of M2 → M1 repolarized macrophages compared to 24 h M1-polarized macrophages (top) and M1 → M2 macrophages compared to 24 h M2-polarized macrophages (bottom). As a baseline for expected global expression differences between M1-polarized samples over time, the purple line in the top panel shows the average JSD between both later M1 controls (collected at 48 h and 96 h) and the 24 h M1 sample. Similarly, the red line in the bottom panel shows the average JSD between both later M2 controls (48 h and 96 h) and the 24 h M2 sample. After switching media, the JSD for repolarized macrophages decreases as M1 → M2 macrophages become more similar to M2-polarized macrophages and M2 → M1 macrophages become more similar to M1-polarized macrophages.

Given that previously polarized macrophages largely revert to an unpolarized state when external cytokine stimuli are removed, we wished to explore how polarized macrophages would respond when exposed to a new cytokine stimulus. To investigate how macrophages respond to a switch in polarizing cytokines, macrophages which were initially cultured with IFN-γ + LPS to induce an M1 state were washed to remove the original cytokines and then cultured with IL-4, and vice versa (Fig. 3A).

Macrophages that experienced a switch in cytokine stimuli adopted a polarized phenotype corresponding to the new cytokine treatment, displaying a similar phenotypic plasticity to the depolarized macrophages. Macrophages first polarized to an M2 state with IL-4, then subsequently repolarized with IFN-γ + LPS to an M1 state exhibit marker gene expression patterns and global transcriptomes typical of M1-polarized macrophages, and vice versa (Fig. 3B,C). Of the 4,732 genes differentially expressed between M1-polarized and M0 macrophages, 76% are also differentially expressed between M2 → M1 repolarized macrophages and M0 macrophages (Fig. S1, S6A & S7). In contrast, 1,300 genes are differentially expressed between M1 and M2 → M1 macrophages, and the majority of these DEGs were less than twofold up- or down-regulated (Fig. S6B). In cells previously polarized to M2, canonical M1 genes *Myd88*, *Nfkb1*, and *Tnf* all equilibrated to levels similar to that of cells maintained in M1 conditions throughout the experiment (Fig. S8A).

Repolarizing M1 macrophages with M2 cytokines revealed a similar pattern: of 3,824 genes differentially expressed between M2-polarized and M0 macrophages, 58% are also differentially expressed between M1 → M2 repolarized macrophages and M0 macrophages, and 2,225 genes are differentially expressed between M2 and M1 → M2 macrophages, with similarly modest fold changes (Fig. S6B). 72 h after the media switch, M2 marker genes *Pparg*, *Retnla*, and *Stat6* were all expressed at similar levels in M1 → M2 cells compared to macrophages maintained in M2 conditions (Fig. S8B). In terms of JSD, the distance between M2 → M1 repolarized macrophages and 24 h M1-polarized macrophages decreases over time as repolarized macrophages adopt more M1 characteristics; similarly, the distance between M1 → M2 macrophages and 24 h M2-polarized macrophages also decreases over time as those macrophages take on more M2 characteristics, although 72 h was not sufficient to completely adopt the M2 transcriptomic profile (Fig. 3D). Taken together, these results suggest that macrophage transcriptomic phenotypes are highly plastic, regulating most genes to reflect their current signaling environment with minimal residual transcriptional “memory”.

### Polarized macrophages exhibit plasticity in chromatin accessibility

**Figure 4:**
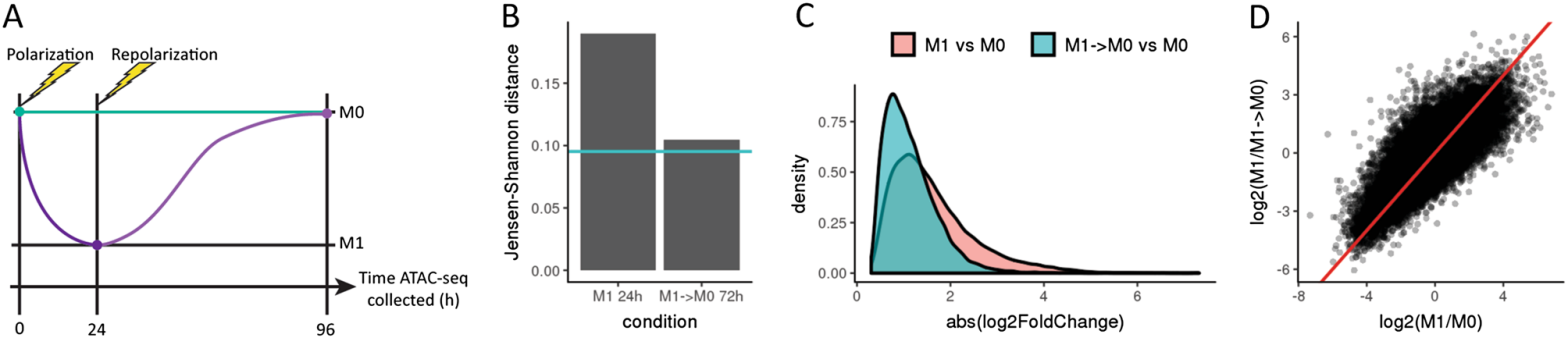
Macrophage chromatin accessibility also reverts to a baseline state when polarizing cytokines are removed. (**A**) Schematic of experimental design, where each point represents a collected sample, and each colored trajectory represents a different treatment condition. Murine bone marrow-derived macrophages were treated with IFN-γ + LPS to induce polarization to M1. After 24 h, cells were washed to remove cytokines and then cultured in base media with no polarizing cytokines for an additional 72 h (n = 3). (**B**) Bar plot depicts Jensen–Shannon distance (JSD) between the ATAC accessibility profiles of polarized and depolarized macrophages compared to the 0 h M0 control. As a baseline for expected global expression differences between samples, the teal line shows the average inter-replicate JSD between the three replicates for the 0 h M0 control sample. JSD values for 24 M1 and 72 h M1 → M0 conditions were computed as the average of JSDs for all unique pairs between the three replicates of each treatment condition and the three replicates of the 0 h M0 control. (**C**) Density plots of log2 fold-change (absolute value) of chromatin accessibility for M1-polarized macrophages and M1 → M0 depolarized macrophages compared to the baseline M0 condition. Median magnitude of log2 fold-change in chromatin accessibility is smaller for the M1 → M0 depolarized macrophages compared to the M1-polarized macrophages. (**D**) Scatterplot comparing log2 fold-change of chromatin accessibility for M1-polarized macrophages versus M0 (x-axis) and M1-polarized macrophages versus M1 → M0 depolarized macrophages (y-axis). The red line (y = x) indicates the expected correlation in the case where M1 → M0 depolarized macrophages are identical to M0 macrophages.

Since our RNA-seq data suggested that macrophage transcriptomes transiently reflect the cells’ current environment, we wished to investigate whether macrophage chromatin accessibility would also be similarly plastic. To this end, we collected bulk ATAC-seq data for three conditions: an unpolarized control (0 h M0), a M1-polarized sample (IFN-γ + LPS for 24 h), and a depolarized M1 → M0 sample (IFN-γ + LPS for 24 h, then basic media for 72 h) (Fig. 4A). Of 8,749 differentially accessible sites between unpolarized (M0) and M1-polarized macrophages, 47% (4,122 sites) remain differentially accessible between depolarized M1 and M0 macrophages. However, the Jensen–Shannon distance (JSD) between the global chromatin profiles of depolarized M1 macrophages and M0 macrophages was comparable to the JSD between the M0 replicates to one another, suggesting that macrophage chromatin profiles revert to a baseline unpolarized state in the absence of extrinsic stimulation and recapitulating trends seen for the transcriptome (Fig. 4B). The median (absolute) log-fold change in differentially accessible sites between depolarized M1 macrophages and M0 macrophages (1.03) was also lower than the median log-fold change between M1 macrophages and M0 macrophages (1.42), indicating that both the number of differentially sites and the magnitude of the accessibility differences at these sites are decreasing as macrophages depolarize (Fig. 4C). Finally, the log fold-change of chromatin accessibility between M1-polarized macrophages and M0 macrophages is closely correlated with the log fold-change of accessibility between M1-polarized macrophages and M1 → M0 macrophages, suggesting that the chromatin accessibility profiles of M0 and M1 → M0 macrophages are broadly similar, despite the presence of differentially accessible sites (Fig. 4D). Looking specifically at sites falling within 500 bp of a gene transcription start site, there does exist a subset of gene-associated sites which remain accessible in M1 → M0 macrophages compared to M0. Of the 10 genes that are fourfold more accessible in M1 → M0 compared to M0, all 10 are also fourfold more accessible in M1 compared to M0 (in total, 132 genes were fourfold more accessible in M1 versus M0; Fisher’s overlapping *p* value = 2.7e−23). Two of these genes are detectably expressed in the bulk RNA-seq timecourse data, but neither exhibits a pattern of upregulated expression in M1 and M1 → M0 (Fig. S9).

## Discussion

Previous studies have established that macrophages are sensitive to changes in their local microenvironment and display a high level of phenotypic plasticity both in vitro and in vivo^2–4^. However, it has remained unclear whether macrophages’ past polarization history left any memory that could impact subsequent repolarization, and whether macrophages, once polarized, could maintain their polarized phenotype without continued extrinsic signals. In this study, we have performed high-resolution transcriptomic profiling of macrophages through polarization to M1 and M2 and then subsequent repolarization (cytokine switch) and depolarization (removal of all polarizing cytokines). These experiments demonstrated that macrophages’ global molecular phenotypes are primarily dependent on the current microenvironment, with little to no memory of past polarization states. Upon initially polarizing cells with M1- or M2-inducing cytokines, they underwent dramatic transcriptional change, traversing a trajectory characterized by the regulation of thousands of genes. However, these molecular phenotypes were transient; upon removal of extrinsic polarizing cytokines, previously polarized macrophages reverted to a baseline state comparable to the unpolarized control, suggesting that continuous external stimulation is required to maintain a polarized macrophage phenotype. Furthermore, we observed extensive repolarization from both M1 to M2 and M2 to M1, suggesting that macrophages may move freely along the phenotypic spectrum between M1 and M2 states. Analysis of chromatin accessibility profiles for M1-polarized and subsequently depolarized macrophages also shows almost complete reversion to baseline, with little evidence of any lingering chromatin memory of the previous polarization state. Most marker genes classically associated with the M1 and M2 phenotypes were expressed at levels that reflected current rather than past signaling, although there were a few genes that had not equilibrated to the levels observed in cells maintained in M1 or M2 cytokines, consistent with a previous study that looked only at such markers^29^. Regardless, Smith et al*.* concluded that a relatively simple regulatory network comprised of feedforward loops to stabilize the M1 and M2 programs and mutual inhibition between them was insufficient to adequately explain their data. Our observations reinforce that conclusion and suggest that the signals tested here, which are classically associated with M1 and M2 macrophage phenotypes, are not sufficient to push cells into stable molecular “attractor” states.

We designed these experiments to maximize the chance of detecting molecular “hysteresis” by using deep transcriptome sequencing with finely resolved temporal sampling under highly controlled conditions. However, there are several important caveats to this design. First, macrophages were treated with high doses of polarizing cytokines; while high doses were used in order to generate a robust polarization response, they may also be responsible for the rapidness of the observed polarization and the lack of asynchronicity between individual cells. The concerted, rapidly transient response we see here may not accurately reflect the responses of macrophages in vivo, where cells could be exposed to a wide array of (potentially conflicting) signals and over a range of doses. Second, these in vitro experiments exclude many co-stimulatory molecules present in the in vivo milieu that, while not classically associated with M1 or M2 phenotypes, may nevertheless stabilize them. Third, it is possible that longer exposure times or alternate doses of these cytokines might elevate cells above a critical threshold of polarization required for hysteresis. Fourth, bone-marrow derived and tissue resident macrophages vary in their response to inflammatory cytokines, so the results observed in this study may not be applicable to tissue resident macrophages. Finally, macrophage polarization varies across genetic background, and macrophages derived from other mouse strains might exhibit different polarization kinetics and potentially attenuated repolarization^30^.

The transience of polarized macrophage phenotypes has implications for potential therapies that rely on reprogramming macrophages. Stabilizing a desired macrophage phenotype may require that cells be continuously exposed to a particular combination of signals or engineered to constitutively activate pathways downstream of those signals. Maintaining a particular signaling milieu in vivo is likely to be challenging after macrophage transplantation, and engineering cells (e.g. with genome editing) carries risks. Nevertheless, understanding the molecular machinery that endows macrophages with such plasticity remains an important goal. That macrophages are so responsive to their current signaling environment suggests that future efforts to dissect macrophage signaling pathways with tissue- and context-specific cues could be very fruitful, both for basic immunology and for clinical applications.

## Materials and methods

### Mice

Wild-type C57BL/6J 6–10 week old mice were obtained from Jackson Laboratory. All experiments were approved by the Institutional Review Board (IACUC, University of Washington) and were performed in accordance with relevant guidelines and regulations.

### Macrophage cell culture

Mice were killed by cervical dislocation, and bone marrow cells were harvested by flushing femur bones with RPMI-1640. Bone marrow cells were plated at a concentration of 6 × 10^6 cells per 3 cm petri dish, then cultured in a base media of RPMI-1640 with 20% horse serum, 100 U/mL penicillin, 100 μg/mL streptomycin, and 20 ng/mL macrophage colony stimulating factor (MCSF). On day 7, cells were stimulated with 100 ng/mL LPS and 25 ng/mL interferon-γ (IFN-γ) or 25 ng/mL interleukin-4 (IL-4) to induce polarization to M1 or M2 states, respectively, or maintained in base media as a control. For repolarization, bone marrow-derived macrophages (BMDMs) were primed with LPS + IFN-γ or IL-4 for 24 h, washed with PBS, then either treated with the opposing stimulus, maintained in the original stimulus, or returned to base media. Samples were collected for bulk RNA-seq analysis 1 h, 2 h, 4 h, 6 h, 12 h, and 24 h post-treatment for polarization, and 1 h, 2 h, 4 h, 6 h, 12 h, 24 h, and 72 h post-treatment for repolarization. Three biological replicates were collected for the full set of polarization and repolarization conditions. For single cell RNA-seq, a single replicate was collected in which cells were harvested and cultured as described above for a subset of polarization conditions: 0 h M0, 6 h M0, 24 h M0, 6 h M1, 24 h M1, 6 h M2, and 24 h M2. For bulk ATAC-seq, cells were also harvested and cultured as described above for three biological replicates of the following conditions: 0 h M0, 24 h M1, and 72 h M1 → M0.

### Sample collection and library preparation

For bulk RNA-seq, samples were harvested by removing the media, then adding trizol directly to the macrophages in the culture plates. Bulk RNA was isolated from the trizol solution via phenol extraction. cDNA synthesis and enrichment were performed using Illumina TruSeq v2 kits on 500 ng total RNA for each sample. ERCC spike-in RNA (Ambion) was added to the total RNA at a final dilution of 1:5,000 according to the ThermoFisher guidelines (1 μL of 1:100 ERCC dilution added to 500 ng of total RNA in a total volume of 50 μL). The libraries were sequenced on the Illumina NextSeq 500 platform using a v2 75-cycle kit (Read 1: 35 cycles, Read 2: 35 cycles, Index 1: 6 cycles). Bulk ATAC libraries were prepared using the Greenleaf protocol^31^ and sequenced on the Illumina NextSeq 500 platform using a v2 75-cycle kit (Read 1: 35 cycles, Read 2: 35 cycles, Index 1: 10 cycles, Index 2: 10 cycles).

For single cell RNA-seq, samples were collected by removing media, washing with PBS, then incubating with 3 mL cold Versene for 5 min on ice. Macrophages were then harvested by scraping the plates. Single cell RNA-seq libraries were generated using 10X v1 and sequenced on the Illumina NextSeq 500 platform using a v2 75-cycle kit (Read1: 98 cycles, Read 2: 10 cycles, Index 1: 14 cycles, Index 2: 8 cycles).

### Read alignments and construction of the expression matrices

Base calls were converted to fastq format and demultiplexed using Illumina’s bcl2fastq/2.16.0.10. Demultiplexed reads were aligned to the mouse reference genome (mm10) using Tophat v2.0.14 with default settings^32^. For the RNA-seq data, aligned reads were quantified using Cuffquant v2.2.2, and then a normalized gene expression matrix was generated using Cuffnorm v2.2.2^28,33–35^. For the ATAC-seq data, PCR duplicates were removed using Samtools v1.9^36^, and data from all alignment files were merged into a single file for peak calling using MACS v2.1.0 (parameters: --nomodel --keep-dup all --extsize 200 --shift -100 -B --SPMR --call-summits) to generate a master list of peaks observed in the experiment^37^. A peak count matrix was calculated by using Bedtools v2.28.0^38^ to compute the intersection between each sample’s aligned reads and the master list of peaks. Expression matrices for the single cell data were generated using the 10X Cell Ranger pipeline (10X Genomics).

### RNA-seq analysis

The normalized gene expression matrix was loaded into R (v3.4.0)^39^ and filtered to include only genes with at least 10 FPKM in at least 1 sample. Multidimensional scaling coordinates were computed based on the average expression values (mean across three replicates) for each sample. Jensen–Shannon distances (JSDs) were computed between each treatment condition and the 0 h M0 control using proxy v0.4-17^39^. Differential gene expression was calculated using Cuffdiff v2.2.2^35^.

For the single cell data, the normalized gene expression matrix was loaded into R (v3.5.2)^39^ and analyzed using Monocle 3 beta (v0.1.2)^40,41^. To generate the UMAP plot, data normalization and preprocessing were run with the default parameter settings, dimensionality reduction and cell clustering were run using the UMAP reduction method option^42^, and graph learning was run using learn_graph_control = list(ncenter = 1,000) and default settings otherwise. Proliferation scores were computed as log10(aggregate_marker_expression), where aggregate marker expression was calculated as the sum of size factor-corrected expression for a panel of cell proliferation marker genes (listed in Fig. S3C). Figures were generated using ggplot2^43^.

### ATAC-seq analysis

The raw peak count was loaded into R (v3.4.0)^39^ and normalized using DESeq2 v1.10.1^44^, then filtered to include only peaks with greater than 10 counts in at least 1 sample. Differentially accessible peaks were also calculated using DESeq2. Sites were considered to be associated with a gene if they were located within 500 bp of a gene’s transcription start site. Using proxy v0.4-17^39^, Jensen–Shannon distances (JSDs) were calculated between the three replicates of the baseline condition (0 h M0) and between each of the replicates for each of the treatment conditions (24 h M1 and 72 h M1 → M0) and each of the 0 h M0 replicates. Final JSD values for each treatment condition were computed as the average of JSDs for all unique pairs between the three replicates of each treatment condition and the three replicates of the 0 h M0 control. Figures were generated using ggplot2^43^.

## Supplementary information

Supplementary figures

## Data Availability

Data generated during the current study will be available upon publication in GEO.
