# Supplementary material for: Trajectory analysis quantifies transcriptional plasticity during macrophage polarization
Source: Sci Rep. 2020 Jul 23;10:12273. doi: 10.1038/s41598-020-68766-w (PMC7378057; doi:10.1038/s41598-020-68766-w)
Supplement: Supplementary file 1 — Supplementary figures [file 41598_2020_68766_MOESM1_ESM.pdf]

Trajectory analysis quantifies transcriptional plasticity during macrophage polarization

Serena X. Liu[1], Heather H. Gustafson[2**], Dana L. Jackson[1], Suzie H. Pun[2,3], and Cole Trapnell[1*]

Author Information:
[1]Department of Genome Sciences, University of Washington, Seattle, WA 98195, United States
[2]Department of Bioengineering, University of Washington, Seattle, WA 98195, United States
[3]Department of Chemical Engineering, University of Washington, Seattle, WA 98195, United States
*Correspondence: coletrap@uw.edu
**Present address: Ben Towne Center for Childhood Cancer Research, Seattle Children's Research Institute, Seattle, WA 98101, United States

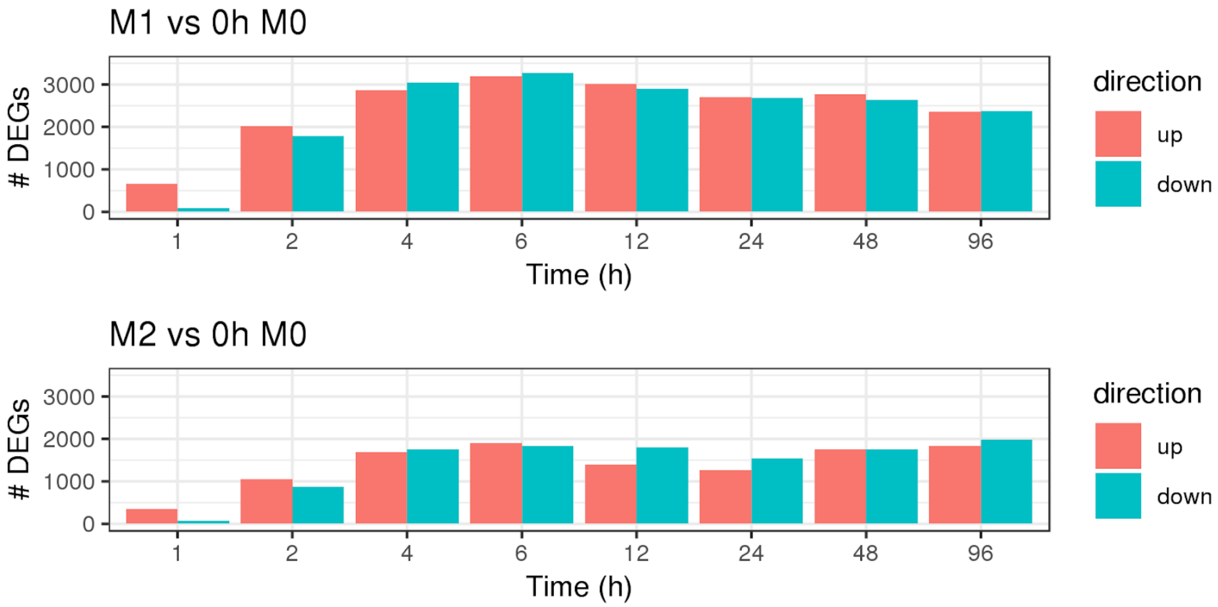

Supplementary Fig. 1. Differentially expressed genes for M1 and M2 compared to 0h M0. Bar plot of the number of differentially expressed genes (up-regulated and down-regulated) with q-value < 0.05 for each M1 and M2 timepoint compared to the 0h M0 condition (n = 3).

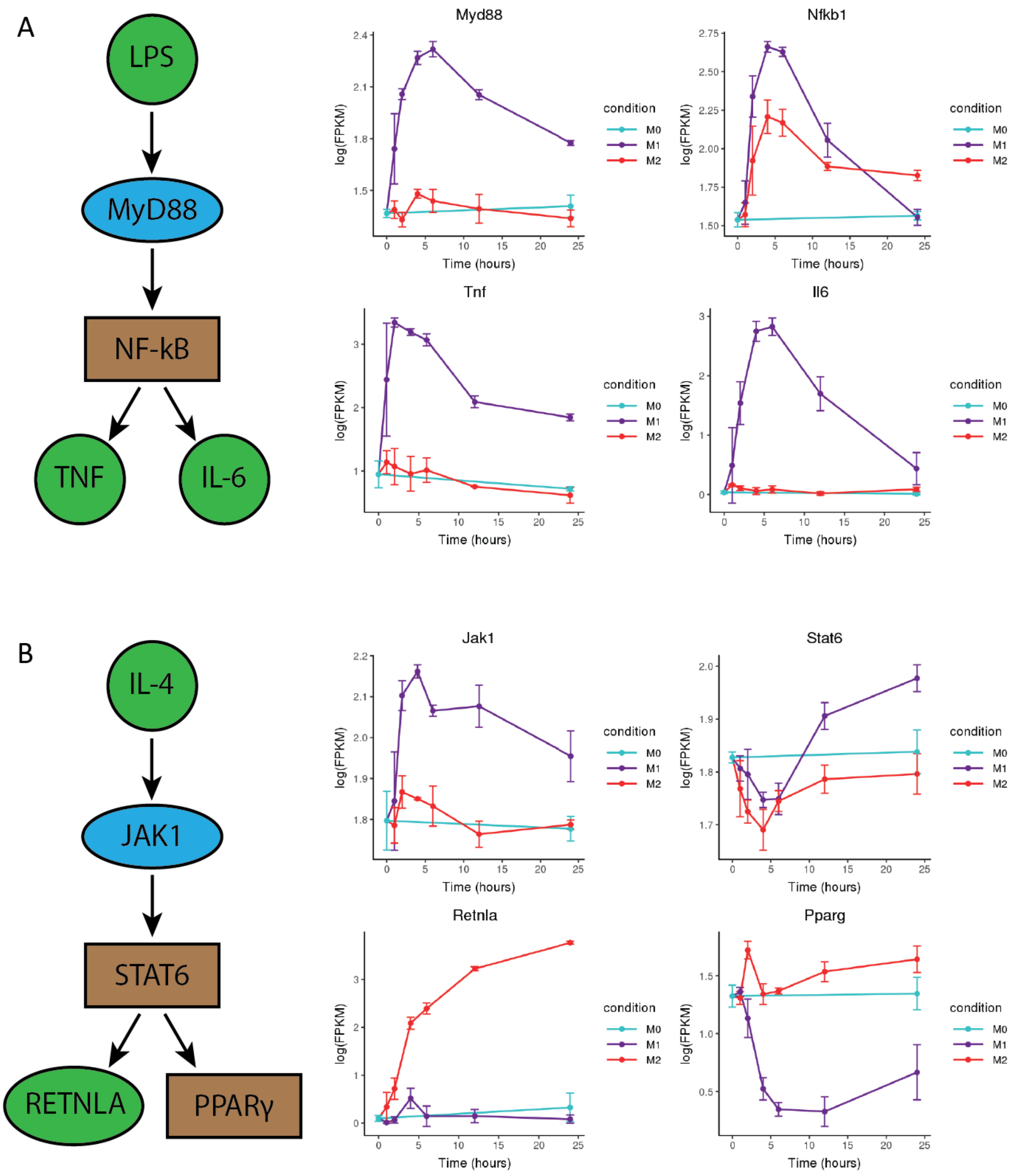

Supplementary Fig. 2. Gene expression of key M1 and M2 marker genes during polarization. A) Schematic of a simplified M1 polarization signaling pathway, highlighting key marker genes (left) and plots of the expression of these marker genes over time during macrophage polarization (right). B) Schematic of a simplified M2 polarization signaling pathway, highlighting key marker genes (left) and plots of the expression of these markers over time during macrophage polarization (right). Some genes, such as *Nfkb1* and *Jak1*, are active in both M1 and M2 polarization, leading to some overlap in expression patterns. Three biological replicates were analyzed for each timepoint.

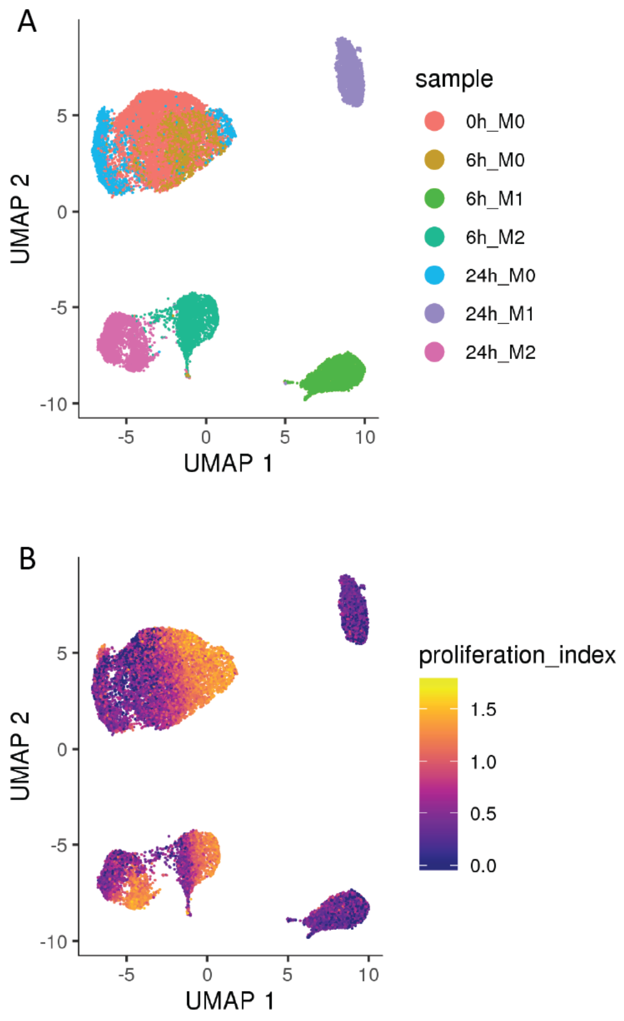

C

Proliferation Markers		
Rpa2	Cks1b	Pcna
Ung	Kif20b	Casp8ap2
Pola1	Kif2c	Rfc2
Blm	Gtse1	Dtl
Gins2	Anln	Gas2l3
Rrm2	Ckap2	Nek2
Cdc45	Cdca3	Hjurp
Gmnn	Cks2	Cenpf
Ccne2	Cdc25c	Ube2c
E2f8	Hmgb2	Birc
Tipin	Rangap1	Aurkb
Brip1	Kif11	Smc4
Mcm4	Anp32e	Cenpa
Dscc1	Ctcf	Cdca8
Cdca7	Kif23	Tubb4b
Mcm6	G2e3	Dlgap5
Mcm5	Bub1	Ect2
Hells	Lbr	Cdk1
Rad51	Mki67	Top2a
Msh2	Ccnb2	Tpx25
Nasp	Ckap2l	Cbx5
Rad51ap1	Nuf2	Tacc3
Wdr76	Ttk	Ncapd2
Fen1	Hmmr	Cdca2
Prim1	Ckap5	Psrc1
Tyms	Tmpo	Usp1
Ubr7	Cenpe	Rrm1
Chaf1b	Nusap1	Clspn
Exo1	Ndc80	Uhrf1
Cdc6	Aurka	Slbp
Mcm2	Cdc20	

Supplementary Fig. 3. *In vitro* polarized macrophages display distinct M1 and M2 phenotypes and synchronous progression. Murine bone marrow-derived macrophages were treated with cytokines to induce polarization (IFN-γ + LPS for M1, IL-4 for M2); M0 was maintained in base media as an unpolarized control (n = 1). A) Macrophages treated with IFN-γ + LPS (M1) and IL-4 (M2) follow distinct polarization trajectories. M1- and M2-polarized macrophages also form distinct clusters by timepoint, showing very little intermixing. B) Cultured BMDMs exhibit low expression of proliferation markers; expression in M1-polarized macrophages is particularly low. Proliferation index is computed as log10(aggregate_proliferation_marker_expression), where aggregate marker expression is calculated as the sum of size factor-corrected marker gene expression for each cell. C) List of the proliferation markers used to compute the proliferation index.

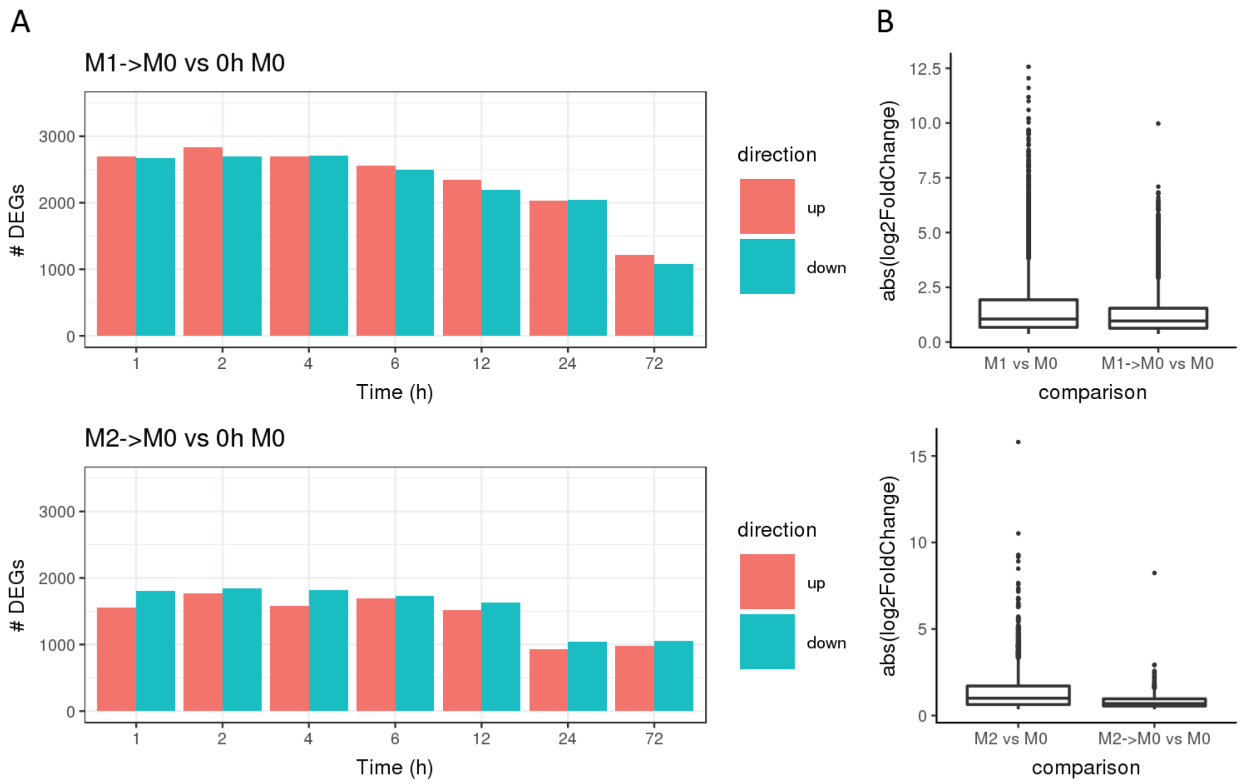

Supplementary Fig. 4. Differentially expressed genes for M1→M0 and M2→M0 compared to 0h M0 and 96h M0. A) Bar plot of the number of differentially expressed genes (up-regulated and down-regulated) with q-value < 0.05 for each M1→M0 and M2→M0 timepoint compared to the 0h M0 condition (n = 3). B) Box plots of the absolute log2 fold change in gene expression of 96h M1 and 72h M1→M0 compared to 96h M0 (top) and 96h M2 and 72h M2→M0 compared to 96h M0 (bottom).

A

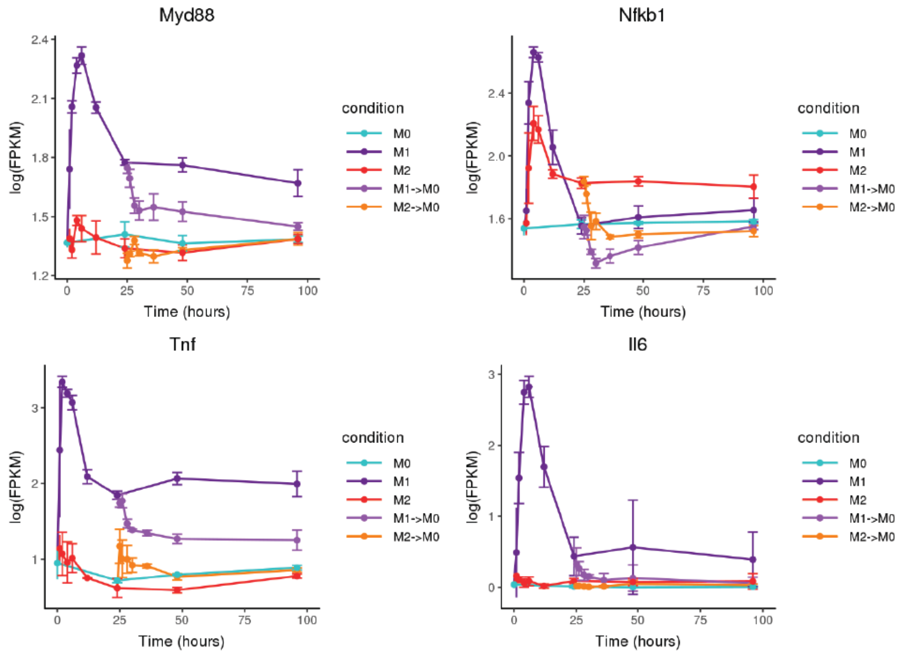

B

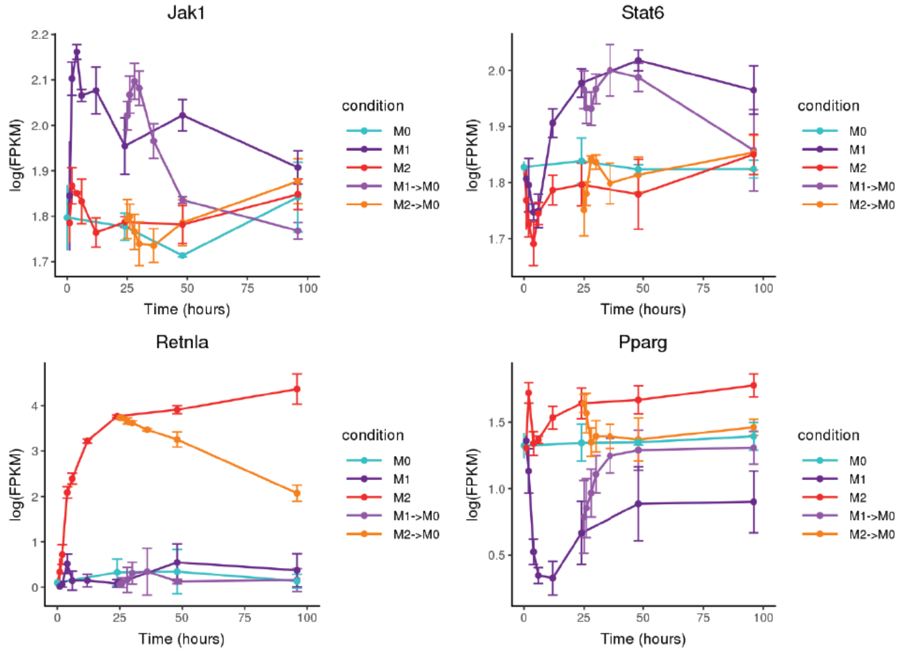

Supplementary Fig. 5. Gene expression of key M1 and M2 marker genes during polarization and depolarization. A) Plots of the expression of M1 marker genes over time during macrophage polarization and subsequent depolarization (n = 3). B) Plots of the expression of M2 marker genes over time during macrophage polarization and subsequent depolarization (n = 3). Some genes, such as *Nfkb1* and *Jak1*, are active in both M1 and M2 polarization, leading to some overlap in expression patterns.

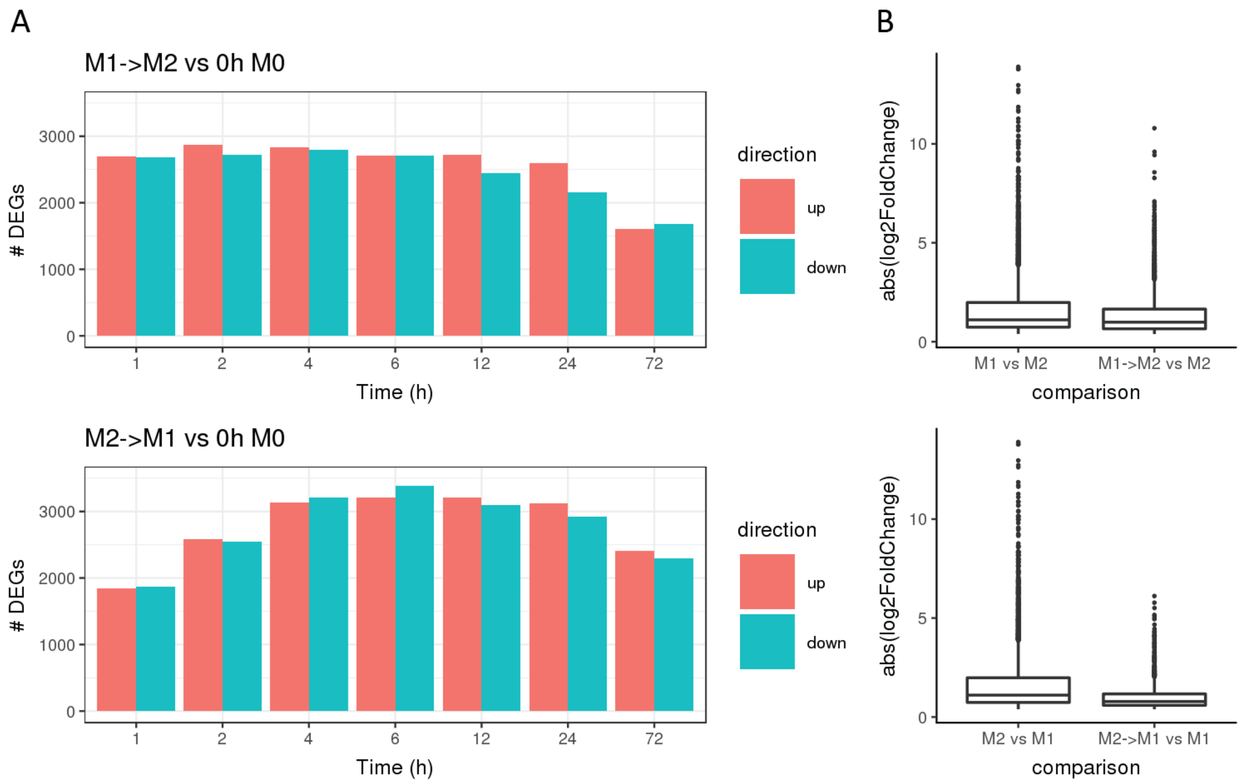

Supplementary Fig. 6. Differentially expressed genes for M1→M2 and M2→M1 compared to 0h M0 and 96h M1 and M2. A) Bar plot of the number of differentially expressed genes (up-regulated and down-regulated) with q-value < 0.05 for each M1→M2 and M2→M1 timepoint compared to the 0h M0 condition (n = 3). B) Box plots of the absolute log2 fold change in gene expression of 96h M1 and 72h M1→M2 compared to 96h M2 (top) and 96h M2 and 72h M2→M1 compared to 96h M1 (bottom).

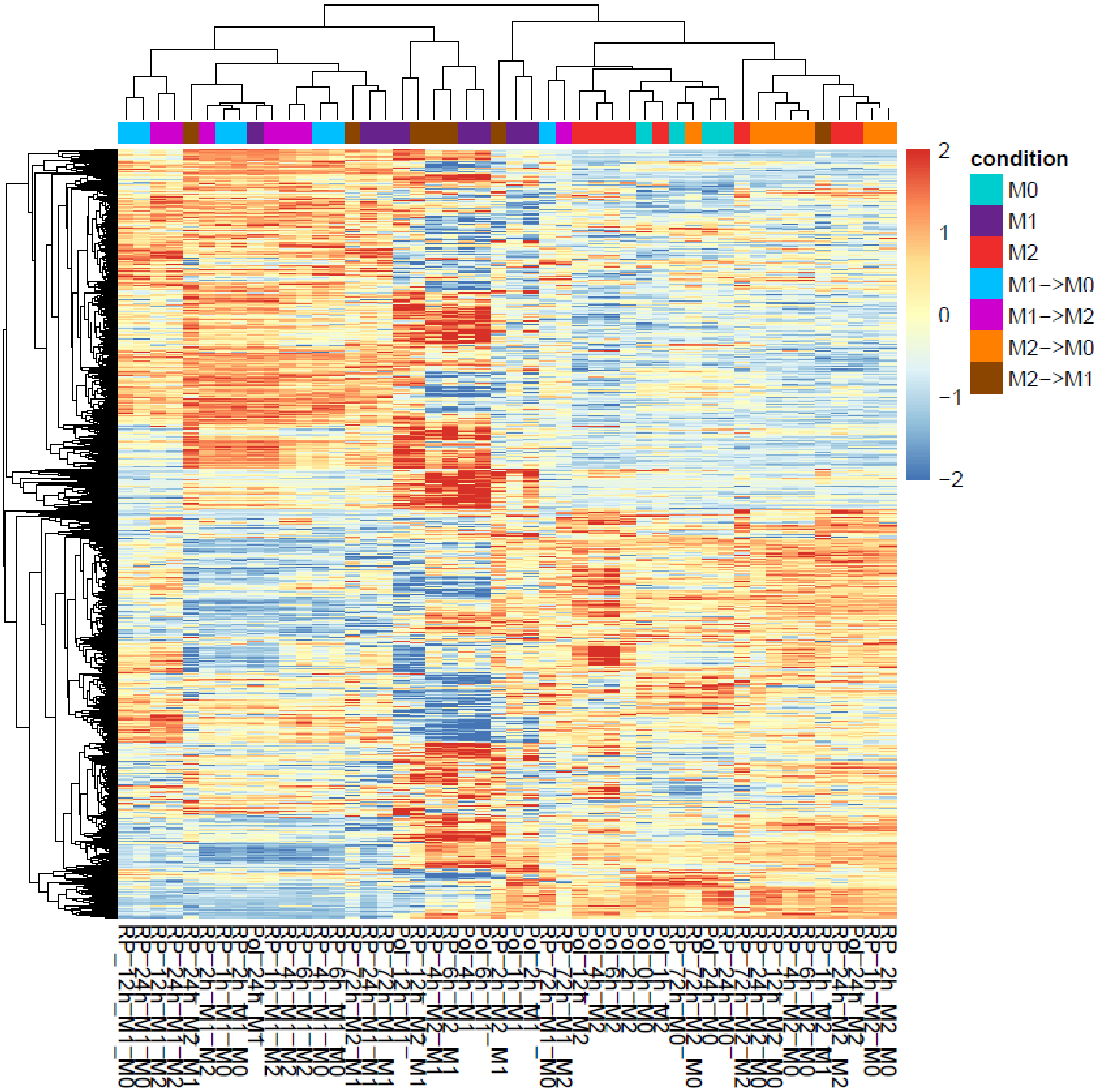

Supplementary Fig. 7. Heatmap of differentially expressed genes. Heatmap of genes differentially expressed between at least one condition and 0h M0 condition with q-value < 0.05, and expressed at a level of >= 10 FPKM in at least one sample (n = 7,460). Rows and columns are clustered using Jensen-Shannon distance, and the heatmap is colored by row z-score. Each heatmap column represents the mean expression across three biological replicates for a sample. Sample labels are structured as follows: <experiment stage>_<time since stimulation>_<polarization condition>_<repolarization condition>. Experiment stage is either “Pol” (first 24h of polarization) or “RP” (subsequent 72h after media switch). Time since stimulation refers to hours since the last cytokine stimulation (either initial polarization or media switch). Polarization condition describes the cytokine treatment for the first 24h of polarization, while repolarization condition (where applicable) describes the cytokine treatment after the media switch. The color coding categorizes samples by the overall polarization/repolarization trajectory.

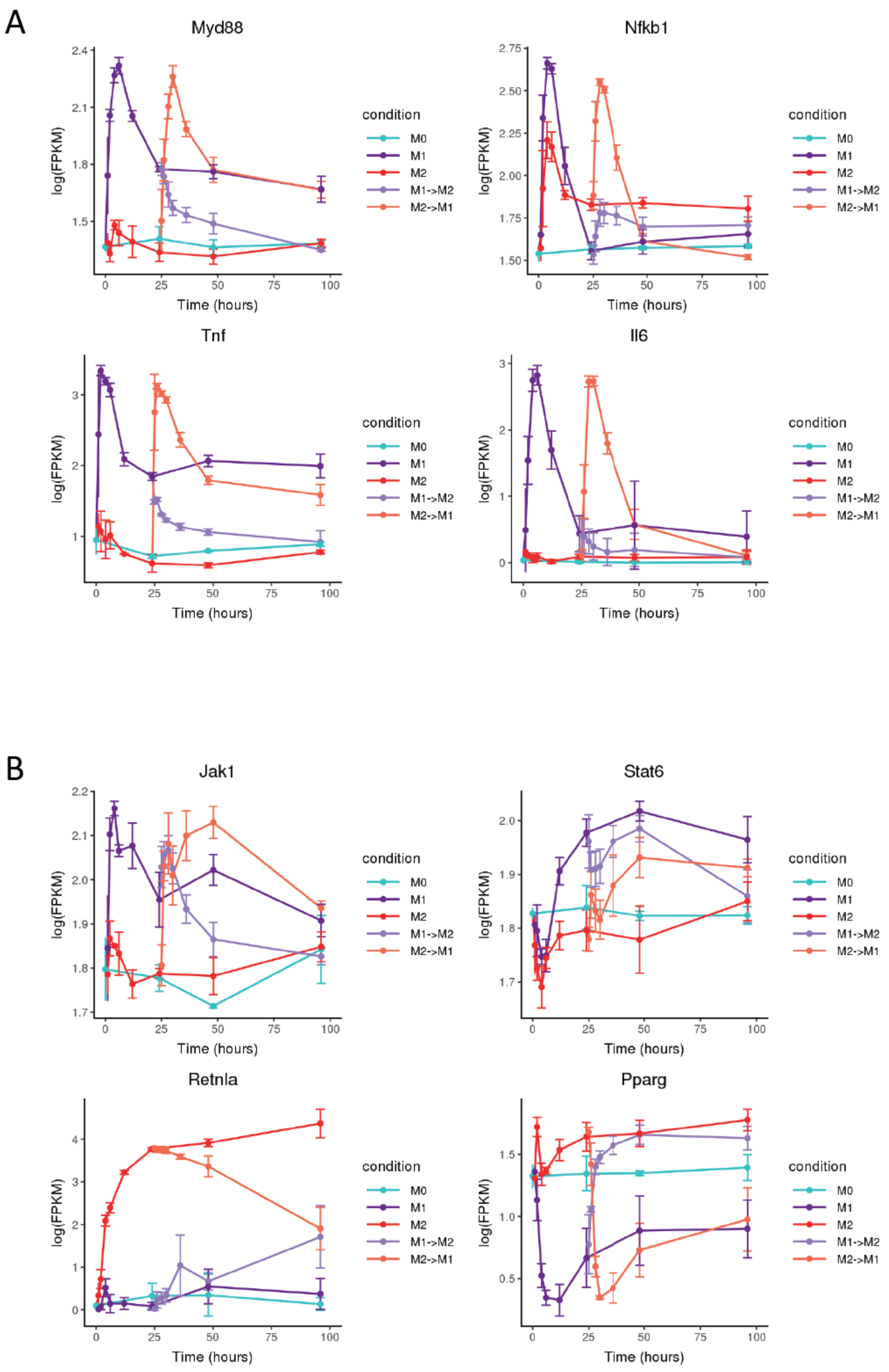

Supplementary Fig. 8. Gene expression of key M1 and M2 marker genes during polarization and repolarization. A) Plots of the expression of M1 marker genes over time during macrophage polarization and subsequent repolarization (n = 3). B) Plots of the expression of M2 marker genes over time during macrophage polarization and subsequent repolarization (n = 3). Some genes, such as *Nfkb1* and *Jak1*, are active in both M1 and M2 polarization, leading to some overlap in expression patterns.

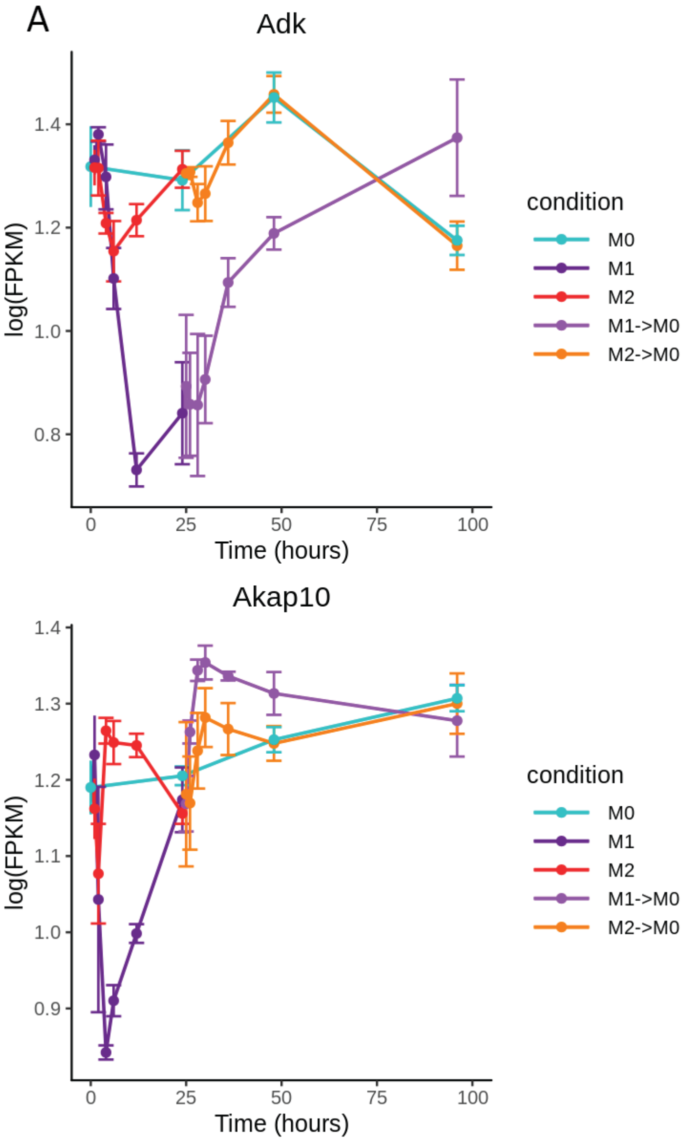

B

M1->M0 vs M0 (4-fold more accessible)
Gm38256
Akap10
Adk
Zfpm
2Spats2l
Gramd1c
Prkg1
Garnl3
Gm43302
Nr3c2

Supplementary Fig. 9. Differentially accessible genes between M1→M0 and M0. Sites which were significantly more accessible in M1→M0 compared to M0 ($q < 0.05$) were further filtered to identify sites which were located within 500bp of a gene transcription start site and which exhibited at least a four-fold change in accessibility. A) Plots of gene expression vs. time for the genes which were linked to four-fold differentially accessible sites and also detected in the bulk RNA-seq timecourse data ($n = 3$). B) Full list of genes linked to sites which were four-fold more accessible in M1→M0 compared to M0. All 10 genes listed were also linked to sites which were four-fold more accessible in M1 compared to M0.
